# Supplementary material for: Content-rich biological network constructed by mining PubMed abstracts
Source: BMC Bioinformatics. 2004 Oct 8;5:147. doi: 10.1186/1471-2105-5-147 (PMC528731; doi:10.1186/1471-2105-5-147)
Supplement: Additional File 2 — The original results of the above study (non-essential files are deleted to keep the file size under the limit set by BMC bioinformatics). [file 1471-2105-5-147-S2.bz2 › chilibotAdditionalFile2/dip05/453ID10477718E10464/html/CCL2_CCR5.html]

 


 **CCL2** and **CCR5** 
  
Found 168 abstracts in PubMed, retrieved 05.  
 

 What does Google say? 
 PDF only 
| .edu only 

---

**Interactive relationship** (e.g. stimulation, inhibition, etc)

**Neutral relationship**- **CCR5**  is a G protein coupled receptor for RANTES, MIP 1a, MIP 1b and MCP  [ **CCL2** ]  2 that functions as the front line coreceptor for human immunodeficiency virus type 1 infection.  Ref: 12837756 J Biol Chem< MedlineTA>J Biol Chem,
- The present findings suggest that MCP 1  [ **CCL2** ] , RANTES, and IP 10 may participate in the pathogenesis of mercuric chloride induced tubulointerstitial fibrosis with mononuclear cell infiltration, via CCR2, CCR1 or  **CCR5** , and CXCR3, respectively.  Ref: 12834626 Exp Mol Pathol, 2003

**Non-interactive relationship** (e.g. studied together, co-existance, homology, etc.)

- Recent studies have demonstrated that SMC possess a number of functional chemokine receptors, including  **CCR5** , CXCR4, and a receptor for monocyte chemoattractant protein 1  [ **CCL2** ]  MCP 1  [ **CCL2** ] .  Ref: 12851644 Microcirculation, 2003
- Whereas the amplitude of calcium mobilization induced by RANTES stimulation was lower in the  **CCR5**  CAM than the wild type WT receptor, MCP 1  [ **CCL2** ]  induced a higher signal in the CCR2 CAM than in CCR2 WT.  Ref: 12837756 J Biol Chem< MedlineTA>J Biol Chem, 2003
